# Intramedullary rod insertion places the femoral component more laterally during Oxford medial unicompartmental knee arthroplasty

**DOI:** 10.1186/s43019-022-00171-1

**Published:** 2022-11-11

**Authors:** Toshikazu Tanaka, Yoshihito Suda, Tomoyuki Kamenaga, Akira Saito, Takaaki Fujishiro, Koji Okamoto, Takafumi Hiranaka

**Affiliations:** 1 Department of Orthopaedic Surgery and Joint Surgery Centre, Takatsuki General Hospital, 1-3-13, Kosobe-Cho, Takatsuki, Osaka 569-1192 Japan; 2 Department of Orthopaedic Surgery, Kobe University Graduate School of Medicine, Hyogo, Japan

**Keywords:** Oxford unicompartmental knee arthroplasty, Intramedullary rod, Bearing dislocation, Wall-bearing space

## Abstract

**Background:**

This study aims to assess the influence of intramedullary rods on the implantation positions of femoral components using Microplasty instrumentation in Oxford unicompartmental knee arthroplasty. We hypothesized that femoral components can be laterally implanted incorrectly when using intramedullary rods.

**Methods:**

This prospective study included all 45 consecutive patients (53 knees) who underwent Oxford unicompartmental knee arthroplasty surgery for anteromedial osteoarthritis or spontaneous osteonecrosis of the knee at our hospital during the study period. A custom-made toolset comprising a triangular caliper and circular trial bearings was used to evaluate the distance between the bearing and the vertical wall of the tibia implant (wall-bearing space) using the caliper at 90° flexion both with and without intramedullary rods.

**Results:**

The wall-bearing space was significantly larger when the intramedullary rod was used than when intramedullary rod was not used (1.8 ± 1.1 mm versus 3.4 ± 1.2 mm, *P* < 0.001). The mean difference of wall-bearing space with and without intramedullary rod was 1.6 ± 0.7 mm.

**Conclusions:**

Femoral components can be laterally implanted incorrectly by an average of 1.6 mm when using intramedullary rods. The wall-bearing space should be evaluated using trial components, and if the relationship is improper, it should be corrected before keel slot preparation.

## Background

Bearing dislocation is a serious complication in Oxford unicompartmental knee arthroplasty (OUKA). The dislocation rate has been reported to be between 0% and 5.7% [1–4]. Several causes of dislocation have been advocated, such as bony or soft-tissue impingement, gap imbalance, and malposition of the components [5]. Bearing contact can cause medial dislocation of the bearing, so the relationship between the bearing and the vertical wall of the tibial component is very important [6]. In addition to dislocation, bearing contact can also induce valgus subsidence of the tibial tray [7]. Meanwhile, bearing separation from the vertical wall allows the bearing to spin, and this could lead to eventual dislocation [8].

**Fig. 1:**
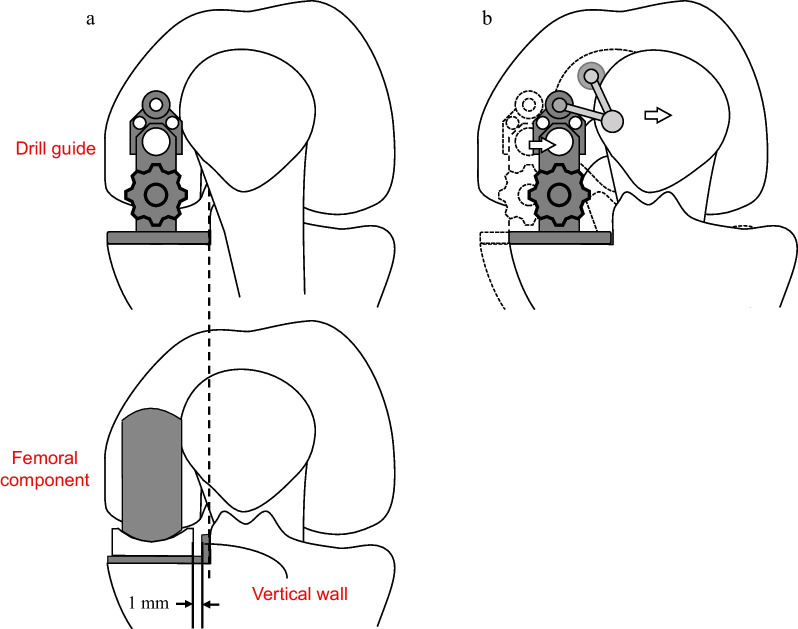
The femoral drill guide of the Microplasty instrument. **a** The width of the bottom of the drill guide is designed to maintain an approximately 1 mm gap between the vertical wall of the tibial component and the bearing. **b** Once the intramedullary (IM) rod is inserted, the IM rod pushes the patella, and eventually the tibia is pulled laterally and the femoral drill guide is placed laterally in relation to the femur

Use of the recently developed Microplasty instrumentation has been reported to result in proper component positioning [9, 10]. One particular advantage of this system is that the placement of the femoral component is dependent on the position of the tibial component. It has been designed to make a 1 mm space between the bearing and the vertical wall, under the condition that the lateral edge of the base plate of the femoral drill guide touches the tibial sagittal cutting plane. Drill holes are then made for the femoral component (Fig. 1a), the positioning of which is extremely important because they decide the femoral component position and, consequently, bearing position.

Despite the design concept behind the Microplasty system, it does not always work properly. Inui et al. [11] reported, for example, that the patella and tibia can be laterally pushed by the intramedullary (IM) rod. Eventually, the drill holes might be made more laterally than planned, and thus the femoral component, along with the bearing, could be implanted more laterally than intended (Fig. 1b). They recommended that the femoral drill guide should be moved medially off the vertical wall, although this has not yet been sufficiently quantitatively assessed. If the effect of use of IM rods on the relationship between the bearing and the vertical wall is quantitatively clarified, medial manipulation of the drill guide to fulfill the ideal relationship between them might be possible. The current study therefore aims to assess the influence of IM rods on the implantation position of femoral components in Microplasty instrumentation for OUKA. We hypothesized that femoral components can be laterally implanted incorrectly when IM rods are used.

## Materials and methods

After obtaining hospital ethics committee approval, we prospectively evaluated all 53 consecutive knees in the 45 patients who underwent OUKA using the Microplasty instrumentation at our institution between April and August 2019 [12]. This study is reported with reference to the Preferred Reporting Items for Systematic Reviews and Meta-analyses (PRISMA) guidelines. Patient demographic data were obtained from a detailed medical chart review (Table 1). All OUKA procedures were performed by an experienced surgeon or under their direct supervision.

**Table 1:** Patient demographic data

Knees/patients	53/45
Sex, female/male	32/13
Diagnosis, OA/SONK	46/7
Age, years	73.8 ± 7.4
BMI, kg/m^2^	25.1 ± 4.1
Preoperative FTA	182.1 ± 3.7
ROM	134.8 ± 15.6
OKS	25.5 ± 7.5
KSS functional	55.6 ± 25.8

Unless otherwise specified, data are given as mean ± standard deviation. *BMI* body mass index, *OA* osteoarthritis, *SONK* spontaneous osteonecrosis of the knee, *FTA* femorotibial angle, *ROM* range of motion, *OKS* Oxford Knee Score, *KSS* Knee Scoring System

Surgical indications for OUKAs included substantial pain, loss of function due to anteromedial osteoarthritis and spontaneous osteonecrosis of the knee (SONK) with intact lateral compartment articular cartilage, and functioning anterior cruciate ligament. SONK was diagnosed by characteristic magnetic resonance imaging and radiography findings [13]. The suitability of OUKA was interpreted using preoperative radiographs referring to the radiographical decision aid, and the final decision was made intraoperatively [14, 15].

### Surgical procedures

All operations were performed according to the manufacturer-provided operation manual [12]. Tourniquet was placed on the thigh, which was supported by a leg holder so that the hip joint was flexed to approximately 30° with the lower thigh hanging in a natural position. The modified under-vastus approach, a kind of subvastus approach, was used in all cases [16]. After removal of osteophytes, a tibial horizontal cut was made with reference to the posterior condyle of the femur using Microplasty instrumentation and a tibial saw guide that had 7° of posterior slope was set parallel to the long axis of the tibia in the sagittal plane. A vertical cut was then performed at the medial tibial spine toward the anterior superior iliac spine.

The IM rod was inserted at 1 cm anterior to the insertion of the posterior cruciate ligament and 2–3 mm lateral to the medial wall of the intercondylar notch into the medullary canal of the femur. The femoral drill guide was inserted so that its base plate made contact with the vertical tibial cutting plane, and then it was linked to the IM rod using the linkage device. The drill holes for the femoral component were made and the posterior condylar osteotomy and gap balancing using the mill were performed as described in the manual [12].

### Measurements of lateral translation of femoral component positions

**Fig. 2:**
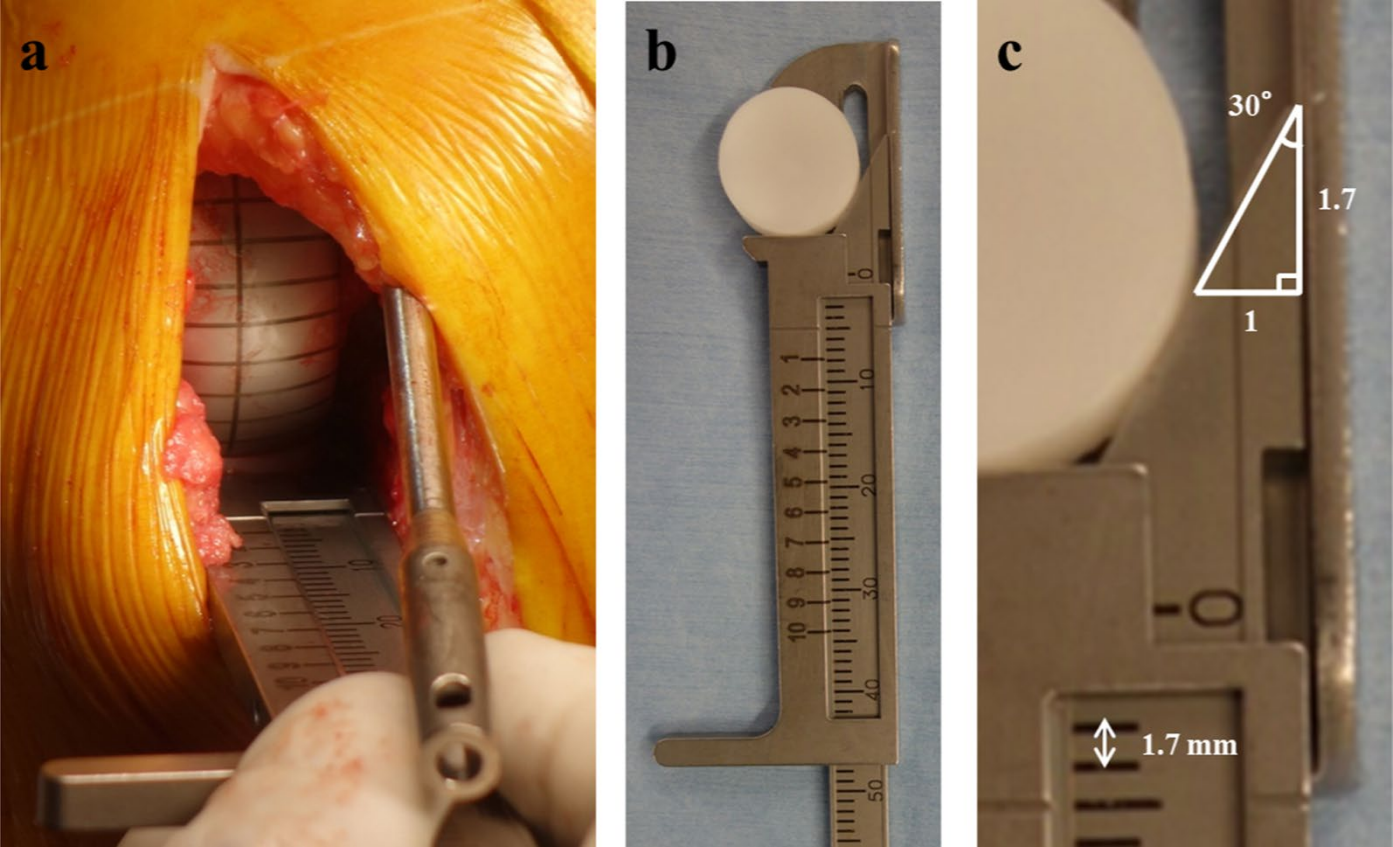
Assessment of the distance between the bearing and the vertical wall of the tibial tray. **a** Measurement of the distance between the bearing and the vertical wall of the tibial tray. **b** Measurement of the distance between the bearing and the vertical wall of the tibial tray using a caliper with the tip in the shape of a right-angled triangle. **c** Caliper with a right-angled triangle shaped tip (30°, 60°, 90°) and a scale of 1.7 mm

**Fig. 3:**
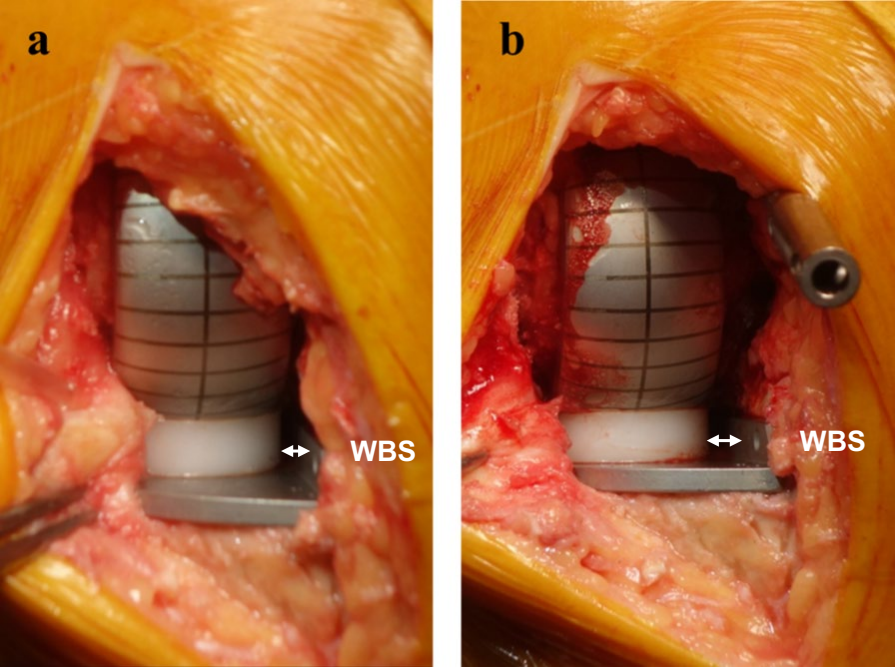
Intraoperative photograph of the two states used in this study: **a** without IM rod, **b** with IM rod

To quantify the lateral translation of the femoral component position, a custom-made toolset comprising a triangular caliper and circular trial bearings was used to evaluate the relationship between the bearing and the vertical wall (Fig. 2). The details of the toolset have been described previously [8, 17]. In brief, to counteract the effect of bearing rotation, the trial has a circular shape with the same diameter and thickness as the actual bearing. Moreover, the circular bearing enables precise measurement of the distance between the bearing and the vertical wall (wall-bearing space, WBS) using the caliper. The WBS was measured at 90° flexion with and then without IM rods (Fig. 3).

### Statistical analysis

To determine the intra- and interobserver reliabilities of the assessments, two investigators performed all assessments twice on 20 randomly selected knees. The calculated sample size in the study by Doros et al. [18] was 20 when two of *κ*, 5% of the alpha levels, and 0.8 of the estimates were demonstrated. The intra- and interobserver reliabilities of all measurements were evaluated using intraclass correlation coefficients (ICCs). The ICCs for intra- and interobserver reliability were > 0.90 (range 0.86–0.95) for all measurements. On the basis of the observed reliability of the results, the measurements taken by a single investigator were used in the current analyses.

A statistical power analysis was performed before this study. A power of 0.8 was expected on the basis of the prespecified significance level of α < 0.05, assuming a medium effect size using G*Power 3. The estimated sample size was 53 knees. Paired *t*-test was used to compare the two states. *P*-values < 0.05 were considered to be statistically significant.

## Results

**Fig. 4:**
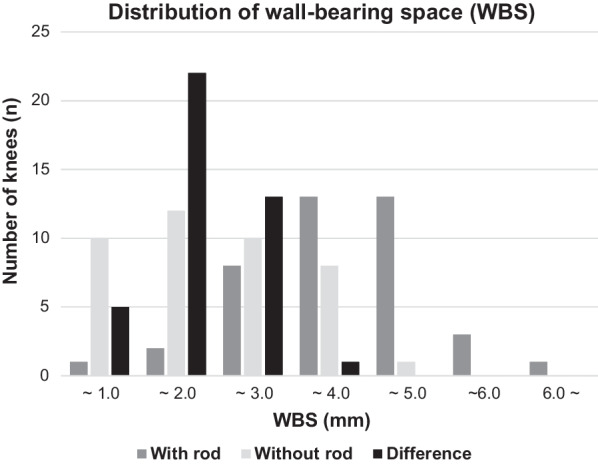
Distribution of the wall-bearing space (WBS) with and without IM rod

The WBS was 1.8 ± 1.1 mm without IM rod, and 3.4 ± 1.2 mm with IM rod. The WBS was thus significantly larger with the IM rod than without the IM rod (*P* < 0.001). The mean difference of WBS with and without IM rod was 1.6 ± 0.7 mm (Fig. 4).

## Discussion

The most important finding of this study was that the WBS increases approximately 1.6 mm with insertion of the IM rod. Once the IM rod is inserted, the IM rod pushes the patella, and eventually the tibia is pulled laterally and the WBS increases. Although change in WBS of 1.6 mm is a small value, it is important to note that the increase in WBS due to the insertion of the IM rod can be corrected intraoperatively. Theoretically, as reported by Hiranaka et al. [8], bearing dislocation due to bearing rotation occurs when the distance between the lowest point of the bearing and the tibial wall exceeds the bottom-corner distance. Conversely, if the ideal femoral and tibial component relationship is fulfilled under the condition of IM rod insertion, the bearing might move close to the vertical wall of the tibia implant, with increasing risk of bearing contact and eventual bearing dislocation and valgus subsidence. These findings support those suggested that the femoral component could be implanted too laterally when using Microplasty in the report by Inui et al. [11], but with a larger sample size. To the best of our knowledge, this is the first study to quantify the effect of the IM rod on the lateral translation of the tibia, leading to an increase of WBS.

Interestingly, an average of 1.8 mm WBS was retained even after IM rod removal. Theoretically, a WBS of 1 mm is expected to be made if IM rod is used, and it will decrease by 1.6 mm when the rod is removed; bearing contact with the vertical wall of the tibia implant should therefore occur in most cases (1.0 mm − 1.6 mm = −0.6 mm). After removal, the WBS was, however, satisfactory or even larger. The manufacturer-provided manual warns that the femoral component should be placed at the center of the femoral condyle [12]. However, this suggested central placement of the femoral component does not always require the base-plate–vertical cutting lane contact because the relationship can change according to the vertical cutting plane, anatomical variation, and the degree of tibiofemoral subluxation. We attempted to confirm the vertical cutting plane contact with the base plate, although it proved to be difficult because of the dark and deep operative field.

**Fig. 5:**
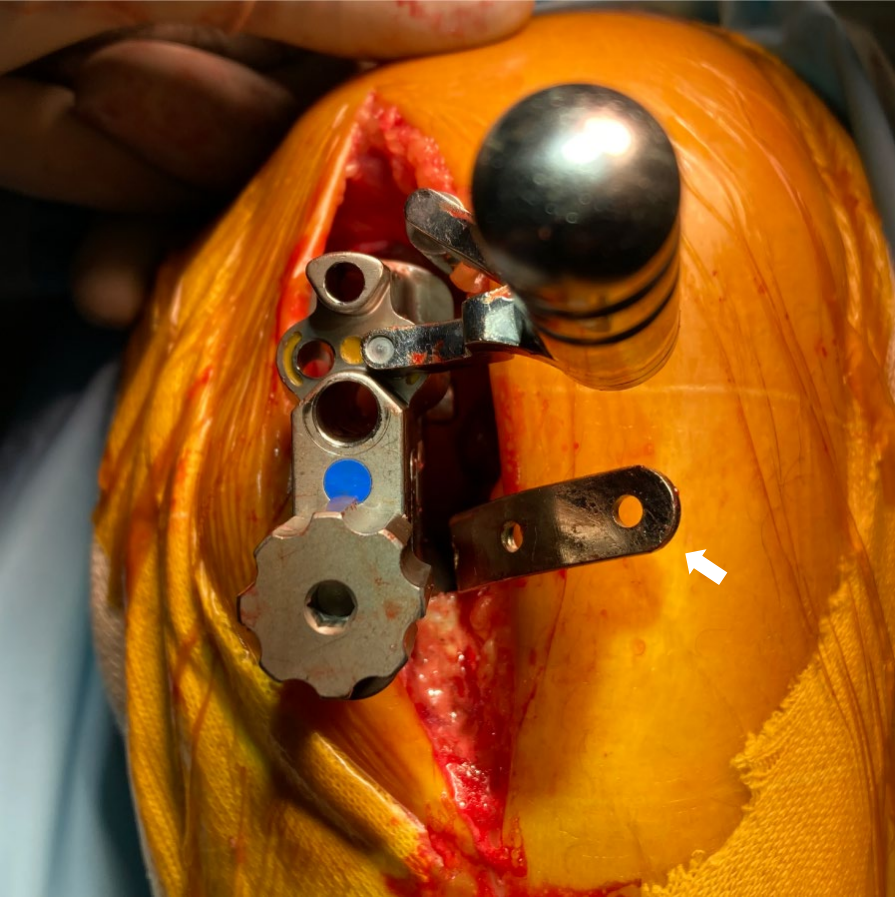
Interposition of the 1.5-mm-width plate. To avoid incorrect femoral component implantations, a 1.5 mm metal plate (white arrow) is inserted between the vertical wall of the tibial component and vertical tibial cutting surface

**Fig. 6:**
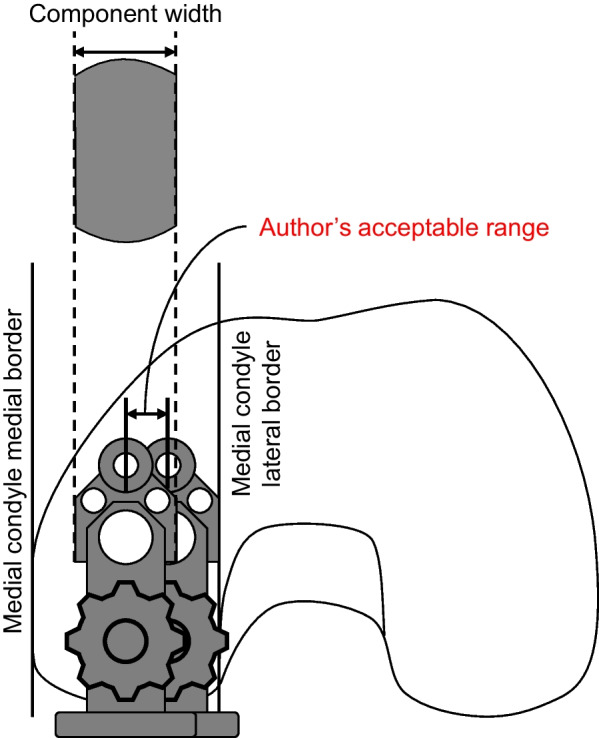
The author’s acceptable range of the femoral drill hole. The author’s acceptable range of the femoral drill hole is between the center of the femoral condyle and the position of the drill hole when the lateral border of the femoral component does not overhang the intercondylar fossa

**Fig. 7:**
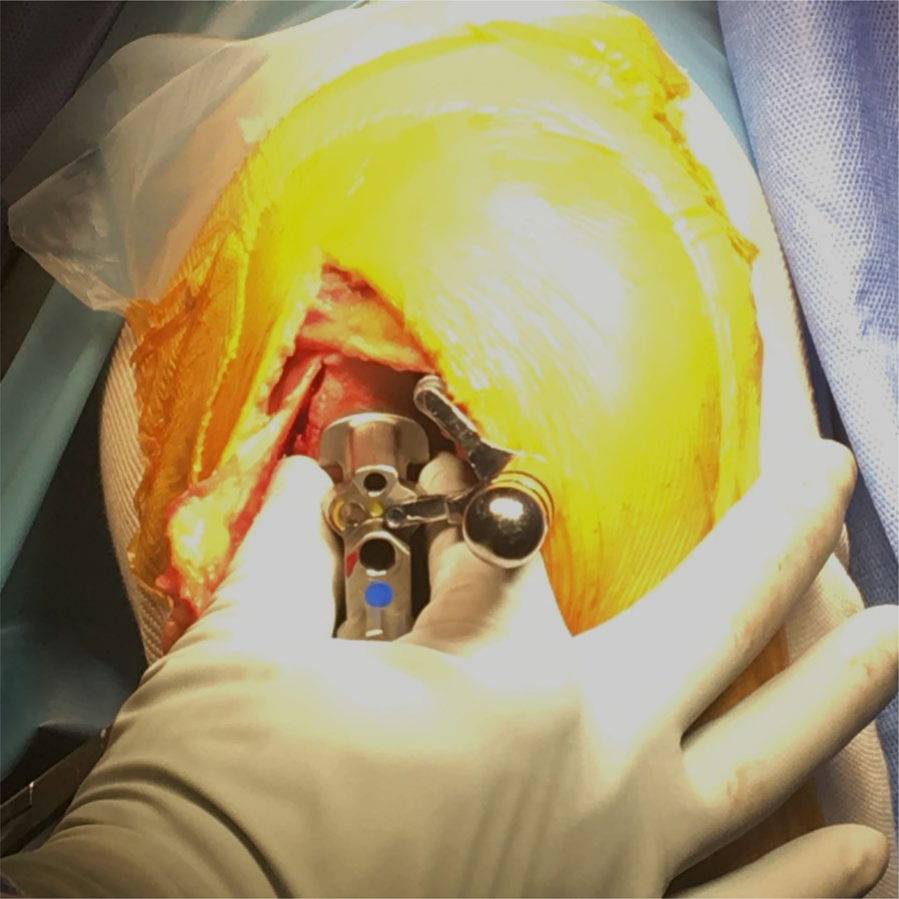
The pinch technique. Operators pinch both medial and lateral borders of the medial condyle using their thumb and index finger to adjust the femoral drill hole position into the acceptable range

Our results also imply that the final WBS cannot be precisely predictable, even with the Microplasty toolset. Although the WBS could be affected by the height and width of the patella, the rotational angle of the sagittal osteotomy of the tibia, and the position of the IM rod insertion, these factors were not evaluated in the present study. Further study is needed to clarify the factors that contribute to the change of WBS. There have been two standards to decide the femoral component position: the vertical cutting plane reference (the lateral edge of the femoral cutting block base plate contacts the vertical cutting plane) and the femoral condyle reference (at the center of the medial femoral condyle), and these standards are sometimes incompatible. We therefore decided the femoral component position considering the results of this study as follows. Firstly, a 1.5 mm metal plate is inserted between the vertical cutting plane expecting the 1.6 mm medial shift of the tibia and the contact among the base plate, the metal plate, and the vertical cutting surface is confirmed (Fig. 5). Secondly, the femoral component position on the medial tibial condyle is evaluated with the knowledge that the width of the drill guide is equivalent to the component width. If it is placed within the “acceptable range”—at the center or slightly laterally but without overhanging—the drill holes are made without any manipulation (Fig. 6). If the drill guide is placed too laterally and there is expected to be overhang, it is manipulated laterally so that the lateral borders of the drill guide and the medial condyle become flush. The “pinch technique” is beneficial, where both borders of the medial condyle are pinched using the operator’s thumb and index finger, along with the drill guide (Fig. 7). Overhanging can be felt by the operator’s fingers, and if it exists, the fingers can be used manipulate it to be flush to the lateral borders. If the drill guide is placed too medially, it is moved to the center of the condyle using the pinch technique. Drill holes are made after the correction of the femoral drill guide position, and the gap adjustments are made. Lastly, the WBS is checked using the trial components before the keel slot preparation. If the trial bearing is in contact with the vertical wall, a sagittal recut is made, as instructed in the manual [12]. If the bearing separates from the vertical wall and is expected to spin, the tibial plate is manipulated medially for the WBS to be 1 mm, as described previously [19]. Using the technique, a satisfactory WBS is fulfilled in most cases. Even if unsatisfactory, an ideal relationship between the bearing and the tibial component can be achieved.

This study has some limitations. Firstly, the alignment of the tibial component, the posterior tibial slope of the medial tibial plateau, the femorotibial angle, and the rotation between the femur and the tibia were not evaluated using radiography or computed tomography. Their measurement and identification of factors that influence WBS are will be undertaken in the near future. Rotational and coronal alignment may have some influence on the amount of WBS. Secondly, this study presents only the results of measurements during surgery. There has been no evaluation of the influence of the lateral translation of femoral components on the impingement of the mobile bearing on the vertical wall of the tibial tray and the valgus subsidence of the tibial components. Thirdly, these measurements were obtained only with the knee in flexion, and different results may be obtained when the knee is in the different position with or without weight bearing. Fourthly, all 53 knees were measured in the same order: without the rod and then with the rod. Bias could therefore affect the measurement result. Finally, surgery was performed using a minimally invasive surgical procedure in all cases. The amount of the lateral translation of femoral component positions may therefore have been larger than that in a case of more invasive surgical procedures.

## Conclusion

When performing OUKAs using Microplasty instrumentation, it is important to recognize that femoral components can be laterally implanted incorrectly by an average of 1.6 mm when using IM rods. The WBS should be evaluated using trial components, and if the relationship is improper, it should be corrected before keel slot preparation.

## Data Availability

The data used to support the findings of this study are included within the article.
